# Consideration of methods for identifying mite allergens

**DOI:** 10.1186/s13601-018-0200-4

**Published:** 2018-04-27

**Authors:** Yubao Cui, Qiong Wang, Haoyuan Jia

**Affiliations:** Department of Clinical Laboratory, Wuxi People’s Hospital Affiliated to Nanjing Medical University, No. 299, Qingyang Road, Wuxi, 214023 Jiangsu Province People’s Republic of China

**Keywords:** Allergen identification, Allergen classification, Dust mite allergen, Mite allergen, Mite allergy

## Abstract

House dust mites are small arthropods that produce proteins—found in their feces, body parts, and eggs—that are major triggers of human allergies worldwide. The goal of this review is to describe the current methods used to identify these allergens. A literature search for allergen identification methods employed between 1995 and 2016 revealed multiple techniques that can be broadly grouped into discovery and confirmation phases. The discovery phase employs screening for mite proteins that can bind IgEs in sera from animals or patients allergic to dust mites. The confirmation phase employs biochemical methods to isolate either native or recombinant mite proteins, confirms the IgE binding of the purified allergens, and uses either in vitro or in vivo assays to demonstrate that the purified antigen can stimulate an immune response. The methods used in the two phases are defined and their strengths and weaknesses are discussed. The majority of HDM-allergic patients may respond to just a small subset of proteins, but new protein discovery methods are still warranted in order to develop a complete panel of HDM allergens for component resolved diagnosis and patient-tailored therapies.

## Background

Sensitization and ongoing exposure to house dust mite (HDM) allergens causes acute reactions including asthma, rhinitis, atopic dermatitis, or other allergic responses [1]. The most common sources of these allergens are the two major HDM species, *Dermatophagoides pteronyssinus* and *Dermatophagoides farinae*, but other dust and storage mite species also contribute [2]. Mite species have differing geographic distributions with *D. pteronyssinus* and *D. farinae* typically overlapping in temperate regions, and with the storage mite *Blomia tropicalis* in the tropics [3].

Almost all body parts of the mites, including the gut, feces, cuticles, and eggs [4–6], contain allergens, triggering allergy in 85% of individuals with asthma. Mite gastrointestinal tract proteins often cause chronic allergy [7, 8] particularly airway allergy, as these proteins are deposited in fecal pellets, which can become airborne and are inhaled. Proteins from the mite cuticle and dermis appear more likely to stimulate atopic dermatitis, as these allergens probably act through skin contact [4]. Importantly, in addition to mite-derived proteins, research is beginning to show potential allergenicity to mite-borne bacteria and fungi, although not to the same level [9].

Over 20 HDM allergens have been characterized to date [10]. The International Union of Immunological Societies (IUIS)—which catalogs allergens by using the first 3 letters of the source of the allergen (e.g., Der for *Dermatophagoides*), the first or second letter of the species (e.g., p for *pteronyssinus*), and finally an Arabic number corresponding to the order in which the allergen was discovered, its clinical significance, or both—has catalogued allergens from four species of dust mites (*D. pteronyssinus*, *D. farinae*, *Euroglyphus maynei*, and *Dermatophagoides microceras*) and six species of storage mites (*Acarus siro*, *B. tropicalis*, *Chortoglyphus arcuatus*, *Glycyphagus domesticus*, *Lepidoglyphus destructor*, and *Tyrophagus putrescentiae*). Proteins from these groups have all been reported to bind (to differing extents) IgEs from serum of patients allergic to dust mites, but the role of most of these proteins in stimulating an allergic response has yet to be defined.

For dust mites, the best characterized allergens are the group 1 and 2 proteins. These represent the so-called major allergens, meaning that the majority of HDM-allergic patients have high levels of high- affinity IgEs directed against these proteins. In a Chinese study of 200 patients, 89% had IgE-reactivity against Der p 1 and 84% had IgE-reactivity against Der p 2 [11]. In an additional study of *D. farinae* extracts, 95% of the patients had specific IgE reactivity against Der f 1 and 95% of patients had IgE reactivity against Der f 2 [12]. The *D. farinae* and *D. pteronyssinus* group 1 and 2 proteins can bind over 50% of the IgEs in HDM-reactive sera (reviewed in [10]). Seven dust mite homologues of the group 1 and 2 proteins are now listed by the IUIS and there are species-specific as well as cross-reactive epitopes [13, 14]. However, for storage mite species, the group 1 proteins are not major allergens; instead the major IgE-binding component in *B. tropicalis* extracts appears to be Blo t 5 [14, 15].

The major dust mite allergens have been well studied [10], hence this review will focus on the techniques used to identify, isolate, and characterize newer allergens. Well-designed discovery and isolation procedures are necessary to establish a complete panel of dust mite allergens for patient-specific diagnostic and therapeutic applications and to provide tools for assessing indoor, outdoor, and occupational environments for mite allergy burden. Ideally, these discovery techniques should be applied to as many mite species as possible. Morgan et al. [16], in assessing human sera for reactivity to *E. maynei*, concluded that this species produces many potent allergens with unique allergenic epitopes, making it “essential that individuals allergic to mites be tested with and treated for all mite species present in the local environment”.

**Fig. 1:**
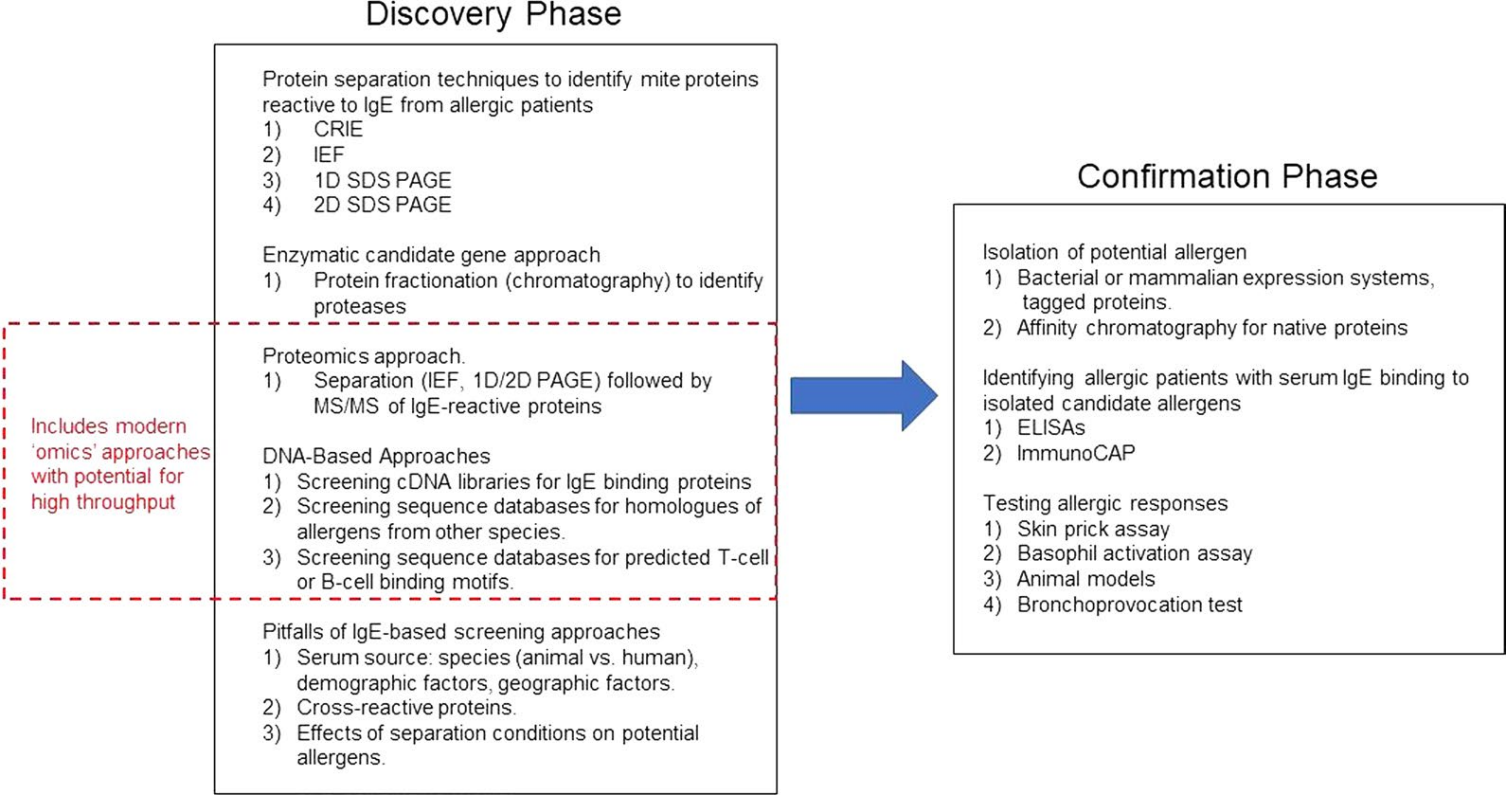
Flowchart summarizing the organization of the review. Identifying allergenic mite proteins typically involves the discovery of a candidate protein (generally based on its ability to bind IgE from sera of allergic patients) followed by confirmation of the isolated protein’s ability to induce an allergic response. The major group 1 mite allergens are serine proteases, which inspired efforts to identify additional proteases with potential allergenicity

In order to stimulate efforts to find new allergens, particularly from lesser- studied mite species as proposed by Morgan et al. [16], this review summarizes the techniques used to discover and confirm the allergenicity of various mite proteins. The structure of the review is shown in Fig. 1. It progresses from the consideration of techniques used to identify candidate allergens to techniques used to isolate and confirm that candidate proteins induce an allergic response.

## Discovery phase

### Identification of potential allergens by IgE binding

Using blood serum samples, researchers are able to identify antibodies, in particular IgEs, resulting from reaction to allergen exposure. It is the most common way to identify an allergic response in humans or animals. Early studies used human sera and crossed radioimmunoelectrophoresis to estimate the number of IgE-reactive species in crude mite extracts. CRIE first separates native proteins based on charge, followed by orthogonal electrophoresis into a gel containing sera where antibody protein precipitates form. Subsequent blotting with radiolabeled anti-IgE reveals reactive peaks. When Chinese HDM allergic patients were screened using this technique, 23 allergen-IgE precipitates were detected in *D. pteronyssinus* and 17 in *D. farinae* extracts [17].

More recent discovery attempts used additional forms of protein separation (isoelectric focusing (IEF), one dimensional sodium dodecyl sulfate polyacrylamide gel electrophoresis (1D SDS-PAGE), two dimensional SDS-PAGE (2D SDS-PAGE), or chromatography) or DNA-based tools (cDNA libraries or genomic approaches) coupled with IgE-binding to identify potential allergens. These studies along with the allergens of interest are found in Table 1 and are displayed chronologically to reveal how the discovery techniques have evolved.

**Table 1:** Methods for identifying mite allergens in the discovery phase

Authors	Methods	PubMed ID	Allergen^a^	Year
Aki et al. [28]	cDNA library screen	7622766	Der f 10	1995
Ferrandiz et al. [18]	1D SDS-PAGE/western blotting	8556562	Der s 1 and 2	1995
Fujikawa et al. [25]	cDNA library screen	8649452	Der f 14	1996
King et al. [6]	Chromatography (protease activity)	8876548	Der p 9	1996
Morgan et al. [16]	IEF, 1D SDS-PAGE/western blotting	9275144	Eur m 2	1997
Wey et al. [53]	Chromatography (mAB)	10592827	94kd IgE binding protein	1997
Le Mao et al. [24]	2D SDS-PAGE/western blotting	9802372	Der f 14, Der f 15	1998
Tsai et al. [61]	Chromatography (mAB)	9723675	Der f 11	1998
Yi et al. [15]	1D SDS-PAGE/western blotting	10697258	Blo t IgE binding proteins	1999
Binder et al. [44]	cDNA library screen	11673567	Plo i 1	2001
McCall et al. [62]	1D SDS-PAGE/western blotting	11292526	Der f 15	2001
Weber et al. [37]	1D SDS-PAGE/western blotting	12847483	Der f 18	2003
Huntley et al. [45]	2D SDS-PAGE/western blotting	15679630	Pso o 10, 11 and 14	2004
Angus et al. [31] and Gao et al. [63]	EST screen	15651897	Blo t 21	2004
Harris et al. [21]	Protease activity screen	15489163	Der p 1	2004
Weghofer et al. [29]	cDNA library screen	18445190	Der p 21	2008
Weghofer et al. [30]	cDNA library screen	23460742	Der p 23	2013
An et al. [27]	Chromatography, 1 and 2D SDS-PAGE/western blotting	23481662	Der f 25, 28, 29, 30	2013
Chan et al. [32]	Sequence mining, 2D SDS-PAGE/western blotting	25445830	Der f 24	2015
Martins et al. [26]	IEF, 1 and 2D SDS-PAGE/western blotting	26015775	Der p IgE binding proteins	2015
Choopong et al. [43]	1 and 2D SDS-PAGE/western blotting	26754146	Aconitate hydrase	2016

^a^If a known allergen was identified in an unbiased screen, this was still considered a discovery attempt

Ferrandiz et al. [18] used 1D SDS-PAGE and western blotting to detect 13 IgE-reactive components from extracts of the poorly studied mite *D. siboney*, a species present in Cuba and recently identified in China [19]. This study went on to purify group 1 and 2 homologues from *D. siboney* by using affinity chromatography with cross-reactive antibodies raised against group one and two proteins from other mite species. Morgan et al. [16] used preparative IEF followed by non-reducing 1D SDS-PAGE and blotting using sera from 16 individual patients to identify 47 potential allergens in the mite *E. maynei*. The percentage of patients with IgEs reactive to a given allergen varied from 6 to 88%, thus helping to prioritize major and minor reactive species. Morgan et al. went on to identify a group 2 homologue by using cross-reactive monoclonal antibodies raised against other group 2 proteins. These studies serve as examples of how candidate gene approaches can be used to identify species-specific homologues of known mite allergens.

Candidate gene approaches based on expected biochemical activity can also be used to identify potential novel allergens. The major group 1 allergens are serine proteases whose enzymatic activities are proposed to enhance the immune response by affecting the barrier function of the mucosa [20]. Based on previous studies suggesting that there were at least three distinct serine proteases in *D. pteronyssinus* fecal extracts, King et al. [6] used chromatography to separate and identify the collagenolytic protease Der p 9 based on its enzymatic activity and weak IgE binding. Additionally, Harris et al. [21] used fluorogenic substrates to perform a large-scale screen for active cysteine and serine *D. pteronyssinus* proteases, but this approach merely resulted in the identification of the known Der p 1 protein indicating that predictions based on proteolytic activity may not be particularly powerful. Alternatively, native and recombinant active proteases may have autolysis activity that interferes with detecting IgE binding [22].

Separation techniques coupled with protein sequencing (a.k.a. proteomics) [23] are capable of identifying a larger range of potential allergens than candidate gene approaches. Le Mao et al. [24] separated *D. farinae* extracts by IEF, 1D, or 2D SDS-PAGE followed by blotting with individual patient sera to estimate the number of IgE-reactive species to be 15–16, 7, or 12, respectively. Known allergens were identified in this screen, including multiple isoforms of Der f 2 and Der f 3 detected using monoclonal antibodies against these proteins. Homologues of Der p 4 and Der p 5 were tentatively identified based on amylase activity (Der f 4) and pI (Der f 5). For antigen discovery, the authors excised two high molecular weight species and microsequenced them with Edman degradation to identify a protein resembling Der f 14 [25] and a protein resembling the chitinase allergen from prawns. A similar study used IEF, ID, and 2D electrophoresis to catalogue multiple IgE-binding *D. pteronyssinus* proteins using sera from allergic dogs [26].

An et al. [27] extended and refined the proteomic approach by first separating *D. farinae* extracts into IgE-reactive fractions using a gel filtration step followed by a clean-up step to remove contaminants that might interfere with 2D SDS-PAGE. 2D-separated proteins were transferred to membranes and probed for IgE-binding activity using pooled sera from HDM-sensitive asthmatic patients. Seventeen reactive protein spots were recovered and identified using electrospray ionization (ESI) quadrupole time-of-flight (Q-TOF) mass spectrometry and found to represent twelve different protein species, of which four were known *D. farinae* allergens. Four (Der f 25, Der f 28, Der f 29 and Der f 30) of the eight novel allergens were purified by gel filtration and ion exchange chromatography. Homogeneity of the purified protein samples was inferred by observing a single IgE-band in blots following 1D SDS-PAGE. Using individual patient sera for western blotting and ELISA, An et al. determined that a high proportion of patients (63–76%) had IgEs reactive to the novel potential allergens. A high proportion of patients (60–70%) also had positive skin prick reactions, and the proteins stimulated a response in a basophil activation assay. This study represents a good example of a how modern biochemical methods have improved antigen discovery and the proteins warrant further analysis as part of the confirmation process, particularly in regards to quantitative measurement of specific IgE titers and environmental levels.

Screening of cDNA libraries has also been used to identify both minor and major allergenic components. *D. farinae* cDNA libraries were screened with HDM-immunized rabbit serum to identify Der f 10 [28] and a high molecular weight reactive protein named Mag3 (Der f 14) [25]. Having a cDNA clone enabled Aki et al. to quickly generate sequence information and produce a recombinant GST-tagged protein for purification and confirmation purposes. Also, once the clone was identified as a tropomyosin homologue, the group was able to use a previously published biochemical purification procedure to isolate the native protein, presumably to a reasonable level of purity. They used dot blots to confirm that approximately 80% of patients had IgEs that recognized native Der f 10 and the major antigens Der f 1 and Der f 2. They used serum from pollen or yeast-sensitive patients as negative controls and followed up the IgE-binding studies with skin prick tests finding that approximately 40% of patients had an immune response to native Der f 10.

More recently, Weghofer et al. screened a *D. pteronyssinus* cDNA library with pooled serum from asthmatic patients to identify new minor (Der p 21) [29] and major (Der p 23) allergens [30]. Again, the cDNA screening approach allowed for rapid production and purification of a recombinant protein, which the authors used in dot blots to examine serum from three different European populations diagnosed with rhinitis, conjunctivitis, and/or asthma. Approximately 70–87% of all patients exhibited Der p 23-specific binding. Using a chip-based Ig-E binding assay demonstrated that only 58% of patients diagnosed with rhinoconjunctivitis had Der p 23-binding IgEs; whereas, 72% of patients with asthma exhibited Der p 23-binding. Quantitation of IgE levels revealed that Der p 23-specific antibodies were present at similar titers to Der p 1 and Der p 2 antibodies, but some patients only reacted to one of the three proteins. They demonstrated the allergenic activity of Der p 23 using a basophil activation assay, confirmed its presence in mite feces, and were able to detect it at low levels in house dust samples. This study is a strong example of a combined discovery and confirmation approach that yielded exciting results.

Other DNA-based approaches, such as whole genome sequencing or cataloguing expressed sequence tags (ESTs), have been used to identify mite allergens but have lagged behind other methods. This is hardly surprising since until recently less than 1% of the potential genomic information from dust mites was publicly available [31]. A draft sequence now allows up to 95% of genes to be identified and provides a scaffold for DNA fragment assembly [32]. The availability of comprehensive sequence data from mites and mite-associated microorganisms have been exploited by using a candidate gene approach to identify species specific homologues of allergens identified from other sources [31, 32]. Chan et al. also coupled their genomic sequencing efforts to 2D SDS-PAGE followed by immunoblotting to identify twelve new *D. farinae* proteins with IgE-binding activity. They used their sequence information to rapidly clone and express six of these twelve candidates and found that only one of the recombinant proteins (Der f 24) bound IgE from the majority of HDM-allergic patients without binding in serum from non-allergic or pollen-allergic controls. Der f 24 was found to induce a positive skin prick reaction in 50% of the tested patients.

A bioinformatic approach was used to mine the human genome to identify substrates for the scabies mite protein Sar s 3 in an effort to identify skin proteins that might be affected by scabies infestation [33]. B and T cell epitopes are less well understood than protease sites, however mite sequences may eventually be mined to identify novel mite allergens based on algorithms used to identify these epitopes. Initial models for linear B cell binding motifs had predictive power only slightly better than random, but newer approaches, particularly ones incorporating multiple models, will likely prove more powerful [34]. Lin et al. [35] used such models to define the molecular characteristics of the allergen Der f 29 for the prediction of four peptides comprising B cell epitopes and five peptides comprising T cell epitopes. This led to the identification of a novel subtype of dust mite allergen, Der f 29b.

In the future, the most powerful discovery techniques will likely combine the genomic and proteomic approaches discussed above, i.e., using protein separation techniques such as 2D gel electrophoresis to identify IgE-reactive components followed by protein sequencing (Edmann Degradation) and now, more commonly, tandem mass spectrometry (MS/MS) (with various peptide fragmentation (e.g., trypsinization), ionization (e.g., ESI, matrix assisted laser desorption), and mass detection techniques (e.g., time of flight (TOF)) in combination with deep RNA or DNA sequencing. Briefly, the differing MS/MS applications generate ionized protein fragments that are categorized by mass and charge and then subjected to a further round of fragmentation and categorization. The resulting mass information of the given fragments is used to identify proteins predicted via the RNA and/or DNA sequencing strategies. These approaches allow for the comprehensive and high throughput identification of multiple IgE-binding proteins from mite body or feces extracts. Recently, this technique was used successfully to identify new IgE binding proteins in both *D. pteronyssinus* and *D. farinae* [36] and could be considered a model approach for use in additional mite species.

### Pitfalls of discovery techniques

As discussed above, there are a wide range of techniques available to discover new potential allergens, and these discovery methods have both general and specific pitfalls. For all screening strategies, it is important to consider the source of serum. Animal models are useful because they can provide a non-limiting supply of reagents and can provide antibodies for later purification purposes. For this reason, early screening methods often used serum from rabbits immunized with whole mite extracts [25, 28] or monoclonal antibodies derived from HDM-sensitized mice to identify potential allergens, which were then confirmed using patient sera. However, this approach can be limiting since animals and humans respond to different mite proteins. For example, the major group 1 and group 2 allergens in humans are not major allergens in dogs [37].

When considering pooling patient sera for screening purposes, it should be noted that patients with different allergic presentations and/or age may exhibit different patterns of IgE reactivity. Of note, allergic symptoms may represent a time course with AD appearing and subsiding in childhood, with appearance of allergic rhinitis and possibly asthma later in life [38]. IgE-binding may also follow a time course.

Geographic differences should also be considered when comparing results of previous studies and when considering new screening efforts. Patients from different geographic areas are exposed to allergens from different mite species and from other invertebrates, and this influences their IgE-binding profiles. Additionally, dust mites, even from the same species, may have regional geographic polymorphisms. Yi et al. [15] reported differences in the IgE-binding patterns from *B. tropicalis* extracts derived from Columbia and Singapore, suggesting possible differences in mite subpopulations. Additionally, naturally occurring variations in IgE binding sites have been identified in Blo t 5 [39].

A particular confounding factor for identifying dust mite causal allergens is the presence of IgEs generated by allergic responses to other invertebrate (i.e., helminth, cockroach, or prawn). This can impact the screening process as allergic patients with low titers of HDM-specific IgEs but high titers of non-HDM-related IgEs can yield false positive binding. This is particularly true for allergens with shared epitopes (i.e., glycosylation) [40]. This issue has raised concerns over whether the group 10 proteins are *bona fide* HDM allergens, as it is known that tropomyosin has IgE-reactive glycosylation sites [41]. Aki et al. [28], who discovered Der f 10, noted that the IgE-binding of recombinant Der f 10 was 25× less than the IgE-binding of the native protein, which could indicate differences in glycosylation as proteins synthesized in *E. coli* lack such modifications. A later study in the same geographic area detected very low titers of recombinant Der f 10-reactive IgEs in asthmatic patients and little activity in a bronchoprovocation test, which indicates that Der f 10 may play little role in respiratory HDM allergies [42]. In contrast, a recent study using 2D SDS-PAGE followed by immunoblotting with sera from patients with allergic rhinitis detected native Der f 10 as a major Ig-E binding species [43]. In this study, 75% of patients had IgEs reactive to Der f 10 while only 50% reacted to Der f 2. The varying results obtained for Der f 10 could be due to differences in the native and recombinant forms of the protein or differing patient populations. For example, Banerjee et al. [4] found that only 10% of asthmatic patients but 67% of patients with atopic dermatitis had recombinant Der p 10-reactive IgEs. Alternatively, patients with high reactivity to group 10 proteins may have been sensitized to tropomyosin homologues from other species [11].

In some cases, cross-reactivity has been used as a tool to identify new dust mite allergens. Binder et al. [44] screened a cDNA library to identify allergens from the Indian meal moth isolating a clone encoding for an arginine kinase (termed Plo i 1). They generated recombinant Plo i 1 in *E. coli* and confirmed its allergenicity using basophil activation and skin prick assays. Intriguingly, they found that their recombinant protein could compete away IgE-binding to related proteins in multiple species, including dust mites. This suggests that arginine kinases are pan-allergens, which in dust mites represent the minor group 20 proteins. This cross-species candidate gene approach is not uncommon in the allergen discovery phase [31] and is often in screens from little analyzed species used as the first step to identify proteins resembling known allergens [45].

For protein-based screens, the source of extracts should be carefully considered as well as any confounding effects based on the chosen separation conditions. Of note, there are differences in the number of allergic components identified in whole mite extracts versus feces-specific extracts [46]. This can limit sensitivity for detecting even known allergens. Choopong et al. [43] separated *D. farina* proteins from whole mite body extracts and detected little Der f 1 because, as the authors claim, this protein is enriched in feces. If the goal of a study is to identify allergens specific for a given condition, appropriate patients and extracts should be used. For example, for asthmatics, it may be more appropriate to consider allergens specifically present in feces since this is believed to be the inhaled component. Additionally, techniques such as IEF, which separates proteins based on charge, and SDS-PAGE, which denatures and separates proteins based on size, have resolutions within specific windows of pI and molecular weight that depend on the chosen conditions. Also, conditions which denature proteins may reduce antibody binding. For example, multiple monoclonal antibodies raised against Der f 1 detected proteins from IEF but not from 1D or 2D SDS-PAGE-separated samples, leading Le Mao et al. [24] to conclude that the antibodies recognize the native protein but not heat denatured forms present in the 1D and 2D PAGE applications. For comprehensive detection of protein species (a.k.a. proteomics) multiple separation techniques should be attempted (see [47]), with the understanding that the number of reactive species can be overestimated due to the presence of multiple isoforms, aggregates, or break-down products of a single expressed protein or underestimated due to separation conditions that affect protein structure and antibody binding.

## Confirmation phase

Screening efforts identify IgE-reactive proteins, but IgE-reactivity is not sufficient to prove that a new allergen has been identified. The confirmation process typically requires purification of native or recombinant candidates, assays (dot blots, western blots, ELISAs or protein microarrays) to determine the percentage of allergic patients that have IgEs reactive to the potential allergen, and use of in vivo or in vitro assays to determine if the purified component can induce an allergic response. Some of these steps are often reported along with the discovery of a new IgE-binding protein (as discussed above), but in many cases the confirmation steps are published in follow-up papers (Table 2).

**Table 2:** Methods for identifying mite allergens in the confirmation phase

Authors	Protein type	Expression system	Method of isolation	% Patients with IgE binding	Test for allerginicity (% Positive)	PubMed ID	Allergen	Year
Ferrandiz et al. [18]	N		Affinity chromatography (mAB)	80–91	NA	8556562	Der s1 Der s2	1995
King et al. [6]	N		Chromatography (protease activity)	92	NA	8876548	Der p 9	1996
Fujikawa et al. [25]	N		Affinity chromatography (AB)				Der f 14	1996
Wey et al. [53]	N		Affinity chromatography (mAB)	37.50	Skin prick (45%)	10592827	Der p 94 kD	1997
Tsai et al. [61, 64]	N		Affinity chromatography (mAB)	> 80	NA	9723675	Der f 11 Der p 11	1998
Olsson et al. [49]	N and R	*E. coli* and Baculovirus	Affinity chromatography (His-tagged)		Basophil activation	9756203	Lep d 2	1998
Kawamoto et al. [5]	R	*E. coli*	Affinity chromatography (GST-tagged)	39	Basophil activation	10381565	Der f 6	1999
Binder et al. [44]	R	*E. coli*	Affinity chromatography (His-tagged)	25	Basophil activation skin prick	11673567	Plo i 1 (group 20 homologue)	2001
Cheong et al. [48]	R	*E. coli*	Affinity chromatography (GST-tagged)	50	Skin prick	12708986	Blo t 3	2003
Ramos et al. [52]	N		Affinity chromatography (mAB)	63	NA	15080814	Blo t 1	2004
Cai et al. [65]	R	*E. coli*	Affinity chromatography (His-tagged)		NA	17639694	Der f 3	2007
Gao et al. [63]	R	*E. coli*	Affinity chromatography (His-tagged)	58.00	Skin prick		Blo t 21	2007
Weghofer et al. [29]	R	*E. coli*	Ion exchange chromatography (no tag)	26	Basophil activation	18445190	Der p 21	2008
Weghofer et al. [66]	R	*E. coli*	Ion exchange chromatography (no tag)	31	Basophil activation	18520154	Der p 5	2008
Beckham et al. [33]	R	*E. coli* and *P. pastoris*	Affinity chromatography (His-tagged) ion exchange chromatography	NA	NA	19812030	Sar s 3	2009
Cui et al. [67]	R	*E. coli*	Affinity chromatography (His-tagged)	NA	NA	19951588	Der f 3	2009
Cui et al. [68]	R	*E. coli*	Affinity chromatography (His-tagged)	NA	NA	20939383	Der f 7	2010
Bordas-Le Floch et al. [50]	N and R	*E. coli*. and *P. pastoris*	Ion exchange chromatography (no tag)	NA	Basophil activation and mouse model	22286395	Der p 2	2012
Weghofer et al. [30]	R	*E. coli*	Hydrophobic interaction and ion exchange chromatography (no tag)	74	Basophil activation	23460742	Der p 23	2013
An et al. [27]	N		Chromatography (gel filtration and ion exchange)	63–86	Skin prick (60–70%) and basophil activation	23481662	Der f 25 Der f 28–30	2013
Banerjee et al. [4]	N and R	*E. coli*	Affinity chromatography (His-tagged or AB)	5–67	NA		Der p 11	2015
Lin et al. [69]	R	*E. coli*	Affinity chromatography (His-tagged)	NA	Skin prick (42.1%) and mouse model	26623108	Der f 27	2015
Chan et al. [32]	R	*E. coli*	Affinity chromatography (His-tagged)	100	Skin prick (50%)	25445830	Der f 24	2015
Cui et al. [70]	R	*E. coli*	Affinity chromatography (His-tagged)	41	NA	26842967	Der f 4	2016
Lin et al. [35]	R	*E. coli*	Affinity chromatography (His-tagged)	NA	Skin prick (24.3%)	27158348	Der f 29b	2016

R: recombinant. N: native. NA: not attempted or too few patients to draw conclusions about prevalence

### Isolation of potential allergens

The majority of papers in Table 2 describe the use of histidine-tagged recombinant proteins synthesized in *E coli* and purified using Ni++ affinity chromatography. GST-tagged Der f 6 [5] and Blo t 3 [48] were purified with glutathione affinity chromatography. The rarity of this approach likely stems from the fact that the GST-tag is bulkier and more likely to interfere with the activity of the purified protein, although the tag can be removed with a standard enzymatic technique [5].

There are few reports of the use of eukaryotic expression systems, e.g. *Pichia pastoris* or *Spodoptera frugiperda* (the baculoviral system), to synthesize dust mite proteins. The touted benefits of these systems include the production of proteins with proper post-translational modifications and proper folding. Misfolding in *E. coli* can lead to the production of insoluble proteins requiring a biochemical refolding step to restore full IgE-biding and/or enzymatic activity. Olsson et al. [49] reported that recombinant Lep d 2 expressed in *E. coli* or in *S. frugiperda* had IgE- binding and basophil activation activities similar to the native protein. Bordas-Le Floch et al. [50] found that recombinant Der p 2 expressed in *E. coli* did have structural differences detected by circular dichroism but had Ig-E binding and basophil stimulation activities comparable to those of the native or recombinant Der p 2 expressed in *P. pastoris*. In contrast, Der p 1 or Der f 1 expressed in *E coli* had significantly less IgE-binding activity when compared to the native form [51]. Hence it is important to consider the structural and post-translational requirements of individual proteins when considering expression systems. For newly discovered allergens, the activity of native and recombinant proteins should be compared.

For isolating native proteins, the most commonly used technique is affinity chromatography using antibodies. Recombinant proteins can be used as antigens to generate specific antibodies which can then be used to isolate the native form [25]. Antibodies can also be generated by immunization with cDNA encoding for the desired target protein [52]. Alternatively, monoclonal antibodies derived by immunizing mice with crude or fractionated mite extracts can serve as tools for purification of potential new native allergens [53]. Additionally, for purification of allergen homologues, cross-reactive antibodies identified in one species can be applied to a new species [18]. In the absence of specific antibodies, alternative chromatography techniques, including ion exchange, gel filtration and hydrophobic exchange, have been used to fractionate whole mite or feces extracts [6, 27, 30, 50]. Fractions were then tested for IgE-binding activity and/or desired enzymatic activity [6] and assayed for homogeneity.

### Testing allergenicity

Once a potential allergen has been isolated, the majority of studies go on to test for IgE-reactivity in individual patient sera drawn from a specific study population. The percentage of patients who exhibit IgE-binding provides a crude measure of whether a protein is a major, mid-tier, or minor allergen. Unfortunately, this aspect of allergen testing has a great deal of variability in terms of techniques used and outcomes reported. Dot blots, western blots, and ELISA have all been used to measure the prevalence of IgE response to a given allergen, but most data in the literature is qualitative and difficult to compare between groups. Allergic patients often exhibit a wide range of total IgE levels which represents antibodies derived from multiple sensitizing proteins found in the environment, and this is worth considering when performing assays with binary outcomes (a.k.a. binding versus non-binding). Quantitative solid phase assays such as ImmunoCAP [54] are easier to compare between groups and can provide a more comprehensive picture of how relevant a given allergen will be for an individual patient. Naturally, the accuracy of these tests (both qualitative and quantitative) are highly dependent on the quality of the purified proteins. Standardization of techniques used for isolation, verification of purity, and quantification of IgE binding should help reduce some of the variation in the field.

Additionally, it should be noted that simply binding IgEs does not indicate that a protein is an allergen. The ultimate test of allergenicity is when a protein can elicit an immune response. Skin prick tests are considered the gold standard for demonstrating sensitivity to a given allergen [54]. Both positive (histamine) and negative (diluent) controls are necessary and a wheal size > 3 mm larger than the negative control is considered a positive result. In general, there is a good concordance between IgE-binding and skin prick responses [55]. However, An et al. [27] reported that 100% of patients had IgE-reactivity to Der f 24, but only 50% were positive in the skin prick test. The other most common used test for allergenicity, is the basophil activation assay where peripheral blood basophils isolated from allergic patients are tested for upregulation of CD203c when challenged with a purified potential allergen.

Skin prick and basophil activation assays are currently the most frequently used tools to verify an allergic response. However, there are additional models relevant for airway allergies. Animal models have been developed which recapitulate aspects of respiratory allergies in humans. Bordas-Le Floch et al. [50] examined T cells isolated by bronchial lavage from Der p 2-sensitized mice demonstrating that recombinant Der p 2 could stimulate cytokine release. They also used an in vivo mouse model of asthma to test the effectiveness of Der p 2 protein immunotherapy. Sublingual treatment of Derp-2 sensitized mice with recombinant Der p 2 significantly reduced airway hyperreactivity as measured by whole body plethysmography. Airway challenge models have also been used in clinical testing. Minami et al. [42] used a bronchoprovocation test to demonstrate that there was a strong correlation between Der p 1 and Der p 2-specific IgE levels and airway response to an HDM-challenge. There are additional clinical assays for allergen testing relevant to rhinitis, conjunctivitis, or other allergic responses [56].

### Assessing allergens in the environment

Confirming an allergic response under laboratory conditions is an important step towards defining an allergen. However, ultimately, the protein should be able to elicit a response under natural conditions, including the concentrations found in the home or work environments. Also, understanding these local concentrations can help lead to the identification of high risk areas and the development of strategies to mitigate exposure.

The growing availability of isolated allergens offers an opportunity for developing assays (particularly ELISA-based assays) that could be used to quantify allergen levels in dust. Yasueda et al. [57] developed a fluorometric ELISA for detecting the major allergen Der p1/Der f1 and used this technique to assay the levels of this allergen on skin and bedding finding values ranging from 1.1 to 354 ng/m^2^. A highly sensitive assay was necessary to detect such low levels, and similar or even greater sensitivity may be required for the detection of other putative allergenic mite proteins. Additionally, airborne concentrations of these allergens, which are likely the most relevant form when considering the development of respiratory symptoms, are even more difficult to detect. It should also be noted that studies using environmental sampling typically report considerable variation even when testing different sites in the same room [58], making it difficult to calculate the precise dose experienced by a given patient. This limits our understanding of how environmental exposure contributes to sensitization and development of symptoms and is a major limitation when attempting to definitively demonstrate the allergenicity of a given protein.

## Conclusion

House dust mites are a major source of indoor allergens [59]. More than 80% of humans with allergies to dust mites have high serum levels of IgE antibodies to the group 1 or group 2 proteins (reviewed in [10]). Additional potential allergens have been identified by screens relying on IgE-binding. Also, advances in proteomic and genomic techniques, in particular the availability of the draft sequence of the *D. farinae* genome, should allow multiple potential HDM allergens to be identified [32]. In fact, a recent review proposed that genome sequencing and metabolomics are the future of allergen discovery and treatment [60]. With these techniques, the bottleneck is likely to be the confirmation step which requires production of high quality purified proteins, well-controlled IgE binding studies, and relevant assays to ensure allergenicity. A complete panel of mite allergens should improve the diagnosis and individualized treatment of patients allergic to these species, and specific and well-designed discovery and confirmation techniques (as discussed in this review) are needed to achieve this goal.
